# Strategic allocation of working memory resource

**DOI:** 10.1038/s41598-018-34282-1

**Published:** 2018-11-01

**Authors:** Aspen H. Yoo, Zuzanna Klyszejko, Clayton E. Curtis, Wei Ji Ma

**Affiliations:** 1 Department of Psychology, New York University, New York, NY USA; 2 Center for Neural Science, New York University, New York, NY USA

## Abstract

Visual working memory (VWM), the brief retention of past visual information, supports a range of cognitive functions. One of the defining, and largely studied, characteristics of VWM is how resource-limited it is, raising questions about how this resource is shared or split across memoranda. Since objects are rarely equally important in the real world, we ask how people split this resource in settings where objects have different levels of importance. In a psychophysical experiment, participants remembered the location of four targets with different probabilities of being tested after a delay. We then measured their memory accuracy of one of the targets. We found that participants allocated more resource to memoranda with higher priority, but underallocated resource to high- and overallocated to low-priority targets relative to the true probability of being tested. These results are well explained by a computational model in which resource is allocated to minimize expected estimation error. We replicated this finding in a second experiment in which participants bet on their memory fidelity after making the location estimate. The results of this experiment show that people have access to and utilize the quality of their memory when making decisions. Furthermore, people again allocate resource in a way that minimizes memory errors, even in a context in which an alternative strategy was incentivized. Our study not only shows that people are allocating resource according to behavioral relevance, but suggests that they are doing so with the aim of maximizing memory accuracy.

## Introduction

One of the hallmarks of VWM is that it is supported by a limited resource. In natural environments, where objects vary in how relevant they are, the process by which our memory resource is allocated appears flexible and strategic. Indeed, experiments demonstrate that increasing the behavioral relevance of a set of items results in better memory for those items^1–5^. Yet, it is still unknown how people decide how much resource to allocate to the encoding and storing of items with different behavioral relevancies.

**Figure 1:**
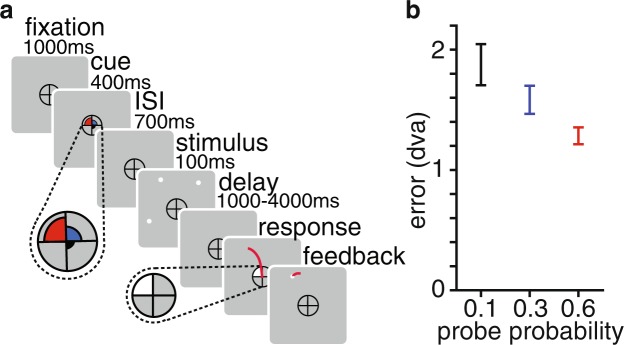
Exp. 1 task sequence and behavioral results. (**a**) Task sequence. (**b**) Main behavioral effects. Estimation error (*M* ± *SEM*) decreases as a function of increasing priority. black: 0.1, blue: 0.3, red: 0.6. dva: degrees of visual angle.

Here, our overall objective is to use computational models of VWM performance to understand the strategy by which memory resource is flexibly allocated when items vary in behavioral relevance. To do so, we first established that the amount of allocated resource is monotonically related to the behavioral relevance, or priority, of memorized items. We used a memory-guided saccade task in which, on each trial, participants remembered the location of four dots, one in each visual quadrant (Fig. 1a). To operationalize resource prioritization, we used a precue to indicate the probability that each dot would be later probed. On each trial, the probe probabilities were 0.6 (“high”), 0.3 (“medium”), 0.1 (“low”), and 0.0. After a variable delay period, one of the quadrants was cued and the participant made a saccade to the remembered location of the dot within that quadrant. For every trial, we computed the Euclidean distance, in degrees of visual angle, between the true and reported target location. We conducted a repeated-measures ANOVA with priority condition as the within-subject variable. In line with our hypothesis, error decreased monotonically with increasing priority (*F*(1.18,15.37) = 10.95, *p* = 0.003), reflecting the intuition that people allocate more resource to a more behaviorally relevant target (Fig. 1b).

**Figure 2:**
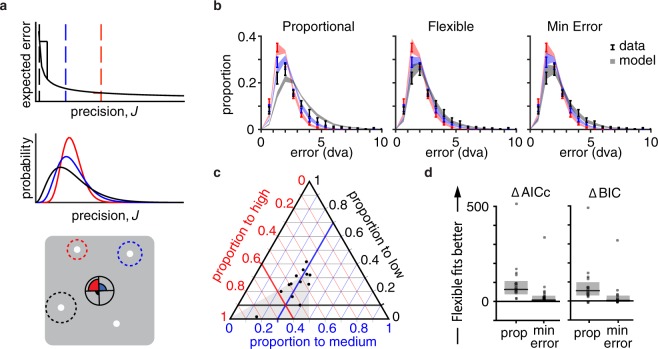
Exp. 1 modeling. Color indicates priority condition – red: 0.6, blue: 0.3, black: 0.1. (**a**) Schematic of the Variable Precision model with a Minimizing Error resource allocation strategy. *Top*, the expected error of a memory decreases nonlinearly with mean precision. In the Minimizing Error model, the amount allocated to each priority item (dashed vertical lines) is determined by minimizing the total expected error (a sum of the expected errors, weighted by their probe probabilities). In this illustration, an observer can drastically decrease their total expected error by allocating some resource from the high-priority item to the low-priority item. *Middle*, the precision *J* of each item on each trial is drawn from a gamma distribution. Items from different conditions, illustrated here in different colors, are drawn from distributions with different mean $\bar{J}$. *Bottom*, the reported location is drawn from a two-dimensional Gaussian with precision *J*. Standard deviation ${J}^{-{\textstyle \tfrac{1}{2}}}$ shown with dotted lines. (**b**) *M* ± *SEM* error distributions for data (error bars) and model predictions (shaded region) for the Proportional, Flexible, and Minimizing Error models (*N* = 14). (**c**) For each participant (black dots), proportion allocated to each priority condition as estimated from the Flexible model. Thicker lines indicate the 0.6, 0.3, and 0.1 allocation to high, medium, and low, respectively. The intersection of these lines is the prediction for the Proportional model. Observers are underallocating to high priority and overallocating to low, relative to the actual probe probabilities. (**d**) Model comparison results. black line: median, grey box: 95% bootstrapped median CI, dots: individual participants. The Flexible model fits significantly better than the Proportional model, but not significantly better than the Minimizing Error model.

Next, we asked what strategy people use to allocate resource in response to unequal relevance. Emrich *et al*.^3^ proposed that resource is allocated in approximate proportion to the probe probabilities. Bays^1^ proposed that resource is allocated such that the expected squared error is minimized. Sims^6^ proposed more generally that resource is allocated to minimize loss. Thus, our second goal was to compare computational models of resource allocation. We tested variable-precision models of estimation errors^7,8^ augmented with different resource allocation strategies. Memory precision for a given item is a random variable whose mean depends on the item’s priority (middle and bottom panels of Fig. 2a; see Supplementary for more a detailed description of the model). In the *Proportional* model, the amount allocated to an item is proportional to the item’s probe probability. This model provided a poor fit to the data (left panel of Fig. 2b), suggesting that people do not allocate resource in proportion to probe probability.

Perhaps this model was too constrained, so we tested the *Flexible* model, in which the proportions allocated to each priority condition were free parameters. We compared models using the corrected Akaike Information Criterion^9^ (AICc) and the Bayesian Information Criterion^10^ (BIC). Both AICc and BIC penalize models with more parameters, but BIC is more conservative. The Flexible model fit the data well (middle panel of Fig. 2b) and formal model comparison showed that it outperformed the Proportional model (median ΔAICc [bootstrapped 95% CI]: 63 [37, 107], ΔBIC: 54 [29, 99]). The proportions allocated to the high-, medium-, and low-priority targets were estimated as 0.49 ± 0.04 (*M* ± *SEM*), 0.28 ± 0.02, and 0.23 ± 0.03, respectively (Fig. 2c), suggesting that the brain underallocates resource to high-priority targets and overallocates resource to low-priority targets, relative to the experimental probe probabilities.

The Flexible model offered a good explanation for *how much* participants were allocating to each item, but not *why*. We considered that resource was allocated to minimize expected loss, where loss is defined as estimation error to a power^1,6,11,12^. In this *Minimizing Error* model, resource allocation differs substantially from the Proportional model^13^. An observer with limited resource should allocate their resource more equally than proportional (Supplementary contains a section showing how optimal allocation changes with total resource). Such a strategy would lower the probability of very large errors for low-priority targets, at a small expense of the high-priority targets (top panel of Fig. 2a). The exponent on the error serves as a “sensitivity to error” parameter: an observer with a large exponent will experience a large error as much more costly than an observer with a lower exponent, and will adjust their strategy accordingly to avoid those errors. The Minimizing Error model fits better than the Proportional model (median ΔAICc [bootstrapped 95% CI]: 49 [21, 99], ΔBIC: 44 [17, 94]. Figure 2b,d) and comparably to the Flexible model (ΔAICc: −7 [−30, −1], ΔBIC: −3 [−26, 3]). Additionally, the model estimated an allocation of resource similar to the Flexible model (0.46 ± 0.02, 0.32 ± 0.01, and 0.22 ± 0.02 for high-, medium-, and low-priority targets, respectively). This suggests that the under- and over-allocation of resources relative to probe probabilities may be rational, stemming from an attempt to minimize error across the experiment.

The first experiment showed that prioritizing items affects memory representations, and that people allocate memory resource in an error-minimizing way. However, this experiment, along with much of the VWM literature, overlooks other information available in VWM: memory uncertainty. Indeed, people can successfully report on the quality of their memory-based decisions^14^, suggesting a representation and use of uncertainty over the memorized stimulus^15–17^. We conducted a second experiment to investigate how, if at all, priority affects working memory uncertainty.

**Figure 3:**
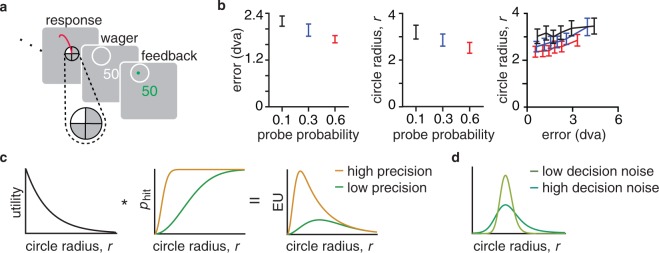
Exp. 2 task, behavior, and model extension. (**a**) Trial sequence. Exp. 2 is identical to Exp. 1 up to the saccade response, after which they make the post-decision wager. (**b**) Main experimental effects. Error bars show *M* ± *SEM* for memory error in degrees of visual angle (dva; *left*) and circle radius in dva (*middle*) across priorities for 11 participants; both measures decrease with increasing priority. These measures are positively correlated within priority conditions (*right*), suggesting that error and circle size have a common cause, namely fluctuations in precision. (**c**) Schematic of how the model generates circle radius predictions. For a given radius *r*, the observer multiplies the utility (*left*) and the probability of the true target being inside of the circle (a “hit”; *middle*) to calculate the expected utility (EU; *right*). Shown here are two examples of how precision *J* effects EU. (**d**) To incorporate decision noise, we model response distribution as a softmax function of utility. Shown here are two examples of how decision noise effects the probability of choosing a particular circle radius.

We tested this with a very similar memory-guided saccade task with an addition wager to measure uncertainty. After the participant made a saccade, a circle appeared centered at the endpoint of the saccade^18^ (Fig. 3a). Participants made a wager by adjusting the size of the circle with the goal of enclosing the true target location within the circle. If successful, they received points based on the size of the circle, such that a smaller circle corresponded to more points. In unsuccessful, they received no points. This procedure served as a measure of memory uncertainty because participants were incentivized to make smaller circles when their memory was more certain.

Our predictions for this experiment were the following: (a) estimation error decreases with increasing priority, (b) circle size decreases with increasing priority, and c) estimation error correlates positively with circle size within each priority level. To test the first two predictions, we conducted a repeated-measures ANOVA with priority condition as the within-subject variable. The ANOVA for circle size violated the assumption of sphericity, so we implemented a Greenhouse-Geisser correction. For the third prediction, we conducted a Spearman correlation for each priority condition, computing correlations across participants as well as for individual participants. For the correlation across participants, we removed any participant-specific main effects by standardizing the data (*M* = 0, *SD* = 1) for each participant before aggregating data for each priority condition.

We confirmed all three predictions. First, estimation error decreased monotonically with increasing priority (*F*(2,20) = 12.5, *p* < 0.001, *η*^2^ = 0.55; left panel of Fig. 3b), indicating that participants allocated more resource to higher priority targets. Second, the radii of the circle wagers decreased monotonically with increasing priority (*F*(1.3,12.9) = 10.60, *p* < 0.005, *η*^2^ = 0.51; middle panel of Fig. 3b), indicating that participants had higher memory certainty in higher priority trials. Third, estimation error and circle size were correlated within each priority level across participants (*ρ*_0.6_ = 0.22, *p* < 0.001; *ρ*_0.3_ = 0.28, *p* < 0.001; *ρ*_0.1_ = 0.18, *p* < 0.001; right panel of Fig. 3b). Correlations at the individual level resulted in similar correlation values (*M* ± *SEM*: *ρ*_0.6_ = 0.22 ± 0.04, *ρ*_0.3_ = 0.27 ± 0.03, *ρ*_0.1_ = 0.16 ± 0.04), though not all correlations were significant. These positive correlations indicate that people have a single-trial representation of their uncertainty independent of the priority manipulation, as suggested by earlier work^15,19^.

This correlation, however, could be driven by some other factor, such as stimulus location or trial delay time. Perhaps stimuli closer to cardinal axes are remembered more precisely than those presented obliquely^20–23^, and knowledge of this effect could be driving the measured within-priority correlation. An effect of delay on error, and knowledge of this, could also be driving the correlation. To test these hypotheses, we conducted two permutations test for each participant and priority level (details in Supplementary). We found that the actual correlations (*M ± SEM:* 0.29 ± 0.04) were significantly higher than the median of the correlations obtained in the null distribution when permuting based on stimulus location (*M *±* SEM:* −0.007 ± 0.006; Wilcoxon signed-rank test, *z* = −4.69, *p* < 1e-5) or delay time (*M *±* SEM:*−0.004 ± 0.004; *z* = −4.53, *p* < 1e-5), suggesting that the correlation within each priority condition was driven by knowledge of internal fluctuations in the quality of the memory representation above and beyond any location- or delay-dependent variation.

We extended the computational models from the first experiment to account for the additional wager data. The observer uses trial-to-trial knowledge of memory quality to calculate the probability that the target lies within the circle of a proposed size. The observer multiplies this value by the utility of that circle size to calculate the expected utility (Fig. 3c). We assume the observer noisily chooses the circle size that maximizes the expected utility (Fig. 3d; details in Supplementary).

**Figure 4:**
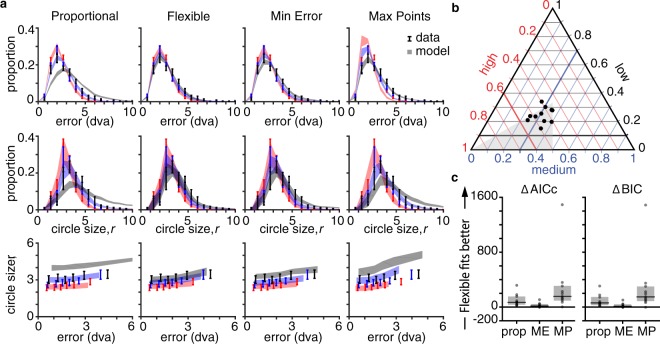
Exp. 2 modeling results. Color indicates priority condition – red: 0.6, blue: 0.3, black: 0.1. (**a**) Fits of four models (columns) to error distribution (*top*), circle radius distribution (*middle*), and correlation between the two (*bottom*). *M* ± *SEM* shown for data (error bars) and model predictions (shaded region). (**b**) For each participant (black dots), proportion allocated to each priority condition as estimated from the Flexible model. Thicker lines indicate the 0.6, 0.3, and 0.1 allocation to high, medium, and low, respectively. The intersection of these lines is the prediction for the Proportional model. Again, observers are underallocating to high priority and overallocating to low, relative to the actual probe probabilities. (**c**) Model comparison results. black line: median, grey box: 95% bootstrapped median CI, dots: individual participants. The Flexible model fits significantly better than the Proportional and Maximizing Points (MP) models, but not significantly better than the Minimizing Error (ME) model.

We again tested the Proportional model and Flexible model, jointly fitting the estimation data and the post-estimation wager data. We again found that the Proportional model did not provide a good fit to human data and the Flexible model provided an excellent fit to the data (left two columns of Fig. 4a). As before, the Flexible model suggests that the brain underallocates resource to high-priority targets and overallocates resource to low-priority targets relative to experimental probe probabilities. The proportion allocated to the high-, medium-, and low-priority targets were estimated as 0.44 ± 0.02, 0.31 ± 0.02, and 0.25 ± 0.02, respectively (Fig. 4b). Given the analogous results, we again asked if we could describe a normative model for the resource allocation strategy.

Unlike in the first experiment, optimal performance in this experiment requires maximizing points. This *Maximizing Points* model has qualitatively different properties from the Minimizing Error model. An observer that maximizes points would receive more points by ignoring the low-priority targets completely in order to remember the high-priority targets better, while an observer that minimizes error would allocate it more evenly across targets. Because these two strategies conflict, we are able to test whether the intrinsically-driven, error-minimizing strategy that people seem to be using in the absence of reward can withstand being put in conflict with an external incentive. To our surprise, the Maximizing Points model fit very poorly (right column Fig. 4a), indicating that participants were not allocating resource in order to earn the most points (Proportional model: median ΔAICc: −75 [−109, −26], ΔBIC: −75 [−109, −26]; Flexible model: ΔAICc: −156 [−308, −94], ΔBIC: −148 [−300, −86]).

Perhaps participants were still acting in accordance with the Minimizing Error model. In this experiment, this strategy is myopic: the observer allocates resource to minimize error in the estimation without considering how this allocation may affect the points of the wager. Nonetheless, the Minimizing Error model fit the data substantially better than the Proportional model (median ΔAICc: 55 [20, 106], ΔBIC: 50 [17, 102]) and the Maximizing Points model (ΔAICc: 140 [85, 249], ΔBIC: 137 [81, 245]) and about as well as the Flexible model (ΔAICc: −16 [−44, −5], ΔBIC: −12 [−40, 0]; third column Fig. 4c). The Minimizing Error model fitted the proportions of resource allocated to high-, medium-, and low-priority targets as 0.52 ± 0.02, 0.32 ± 0.01, and 0.16 ± 0.01, respectively, similar to the allocation estimated in the Flexible model.

In this work, we examined how people allocate resource in a task with items of varying behavioral relevance. First, we found that people flexibly allocate resource according to behavioral relevance. This is comforting: we remember more important things better. Second, we found accurate knowledge of both priority-driven and spontaneous trial-to-trial fluctuations in memory quality, even when controlling for spatial location and delay time. This is also comforting: to some extent, we can trust our confidence in our memories. This knowledge is useful when deciding whether to use or resample information. Third, we explained not just how people allocate VWM resource, but what strategy they may be using. We find that people minimize estimation error, a strategy whose consequence is an overallocation to low- and underallocation to high-priority targets relative to probe probabilities. Fourth, this strategy persists even when presented with a conflicting external reward.

Perhaps we find minimizing the errors of our memory intrinsically rewarding. Rewards influence the metrics of saccades in humans and monkeys^24,25^. For instance, extrinsic rewards affect both the velocity of saccades as well as neural activity in dopamine-associated reward circuits^26^, and they modulate neural activity in cortical areas that represent the goals of saccade plans^27^. Perhaps the intrinsic reward associated with veridical memory eclipses the extrinsic reward associated with gaining more points, which would explain why people minimized error instead of maximized points in the second experiment. Additionally, minimizing memory error might be computationally easier than maximizing points because it does not require the observer to think and optimize performance two steps ahead. In other words, the amount that performance may improve from maximizing points may not be worth the computational and metabolic cost. Future studies should investigate if resource allocation abides by the Minimizing Error model across a variety of experimental probe probabilities (see Supplementary for how optimal resource allocation should change) and reward contexts (e.g., monetary reward^2^).

Our results identify a single, simple model of how the resource that supports VWM is allocated despite the large variability in WM abilities across individuals and age^28,29^. For example, electrophysiological signals measured at the scalp predict individual differences in WM capacity^30^ as well as trial-to-trial variation in the precision of WM^31,32^. Individual differences in WM can also be explained by differences in control processes, such as inhibition of irrelevant distractors^33^. Our model accounts for these individual differences by explicitly assuming inter-trial variability, as well as having parameters that account for participants’ differences in total amount of resource as well as sensitivity to error. Some participants prefer making a few large errors in order to maximize the number of extremely precise memory guided saccades, while others prefer avoiding large errors at the expense of those precise saccades.

While we use the Variable Precision model to study resource allocation in this paper, we do not make any claims about whether this model is the best computational model to capture the data. Its assumptions regarding resource allocation are less constrained, but alternative models such as an Interference^34^ or the Slots Plus Averaging^5^ model may still provide a good fit. Future research could investigate these models as well.

In everyday life, we are bombarded with information constantly, and we have to decide what to look at, pay attention to, and remember. This study finds that not only do people remember more important items and their associated uncertainty, but they also do so in a way that minimizes the overall magnitude of memory errors. Perhaps this strategic allocation is how we are able to function so well despite such limited working memory resource.

## Methods

### Participants

Fourteen participants (5 males, mean age = 30.3, *SD* = 7.2) participated in Experiment 1 and eleven (5 males, age = 28.6, *SD* = 3.03) in Experiment 2. Everyone had normal or corrected-to-normal vision and no history of neurological disorders. Participants were naive to the study hypotheses and were paid $10/hour. We obtained informed, written consent from all participants. The study was in accordance with the Declaration of Helsinki and was approved by the Institutional Review Board of New York University.

### Apparatus

Participants were placed 56 cm from the monitor (19 inches, 60 Hz), with their heads in a chinrest. Eye movements were calibrated using the 9-point calibration and recorded at a frequency of 1000 Hz (Eyelink 1000, SR Research). Target stimuli were programmed in MATLAB (MathWorks) using the MGL toolbox (Gardner Lab, Stanford) and were displayed against a uniform grey background.

In Experiment 2, participants made behavioral responses using a space bar with their left hand and a circular knob (PowerMate, Griffin Technology) with their right hand. For eye-tracking, we applied an online drift correction when the recorded location of center of fixation exceeded 1 degree of visual angle from the center of the fixation cross. This correction was implemented to remove the participants’ discomfort from discrepancies between the actual and measured location of fixation, because this experiment provided live visual feedback of the participants’ current fixation. The online drift correction was not implemented in Experiment 1 because there was no feedback; the corrective saccade provided a measure of drift, which we used offline to correct position.

### Trial procedure

Each trial (Fig. 1a) began with a 300 ms increase in the size of the fixation symbol, an encircled fixation cross. This was followed by a 400 ms endogenous precue, consisting of three colored wedges presented within the fixation symbol, each of which angularly filled one quadrant. The radial sizes and colors (pink, yellow, and blue) of the wedges corresponded to probe probabilities of 0.6, 0.3, and 0.1, respectively. The quadrant with a probe probability of 0.0 did not have a wedge.

The precue was followed by a 700 ms interstimulus interval, then by the targets, presented for 100 ms. The targets were four dots, each in separate visual quadrants. The dots were presented at approximately 10 degrees of visual angle from fixation, with random jitter of 1 degree of visual angle to each location. The location of the targets in polar coordinates were pseudo-randomly sampled from every 10 degrees, avoiding cardinal axes.

This was followed by a variable delay, chosen with equal probability from the range between 1000 and 4000 ms in 500 ms increments. A response cue appeared afterward, which was a white wedge that filled an entire quadrant of the fixation symbol. Participants were instructed to saccade to the remembered dot location within the corresponding quadrant of the screen. If participants took shorter than 100 ms or longer than 1200 ms to make the saccade, the trial was discarded.

In Experiment 1, after the saccade, the actual dot location was presented as feedback and the participant made a corrective saccade to that location. After 500 ms, the feedback disappeared, participants returned their gaze to the central fixation cross, and a 1500 ms inter-trial interval began.

In Experiment 2, simultaneously with the response cue, a red dot appeared at the location of the participants’ fixation as measured online by the eye tracker. Because of eyetracker noise, the red dot occasionally appeared in a slightly different location than where the participant was fixating. In these cases, participants were instructed to adjust their gaze such that the red dot was at the remembered location, and press the space bar to indicate that this was their intended saccade endpoint. After completing this response, participants performed a post-estimation wager. A circle appeared, centered at the saccade endpoint. Participants received points based on the size of the circle, such that a smaller circle corresponded to more points. However, participants were only rewarded points if the true target was within the circle. The number of points awarded was 120*e*^−0.4*r*^, in which *r* was the radius of the circle.

### Data Processing

Processing and manual scoring of eye movement data was performed in an in-house MATLAB function-graphing toolbox (iEye). Eye position and saccadic reaction time (SRT) were extracted from iEye. Statistical analyses were performed in MATLAB (Mathworks) and SPSS (IBM). We excluded trials in which (a) participants were not fixating in the middle of the screen during stimulus presentation, (b) saccades were initiated before 100 ms or after 1200 ms after the response cue onset, (c) pupil data during the response period were missing, or (d) participants made a saccade to the wrong quadrant, ignoring the response cue. This resulted in removing between 1% and 7% of trials per subject. Raw gaze positions were transformed offline into degrees of visual angle using a third order polynomial algorithm that fit eye positions to known spatial locations and we used gaze velocity trace to determine the onset and offset of saccades with a 30°/s threshold. Priority effects were not significantly different between initial and final saccade position, so we report the results for the final saccades.

### Model fitting, Parameter Recovery, and Model Recovery

For each participant and each model, we estimated the parameters using maximum-likelihood estimation. The likelihood of the parameters are defined as *p*(data|model, θ), in which θ is a vector of the model parameters. To calculate the parameter likelihood, we use numerical integration to marginalize over the internal variables **x** and *J* (Supplementary Information). To find the maximum-likelihood parameter estimate, we used the optimization algorithm Bayesian Adaptive Direct Search^35^ in MATLAB, which combines mesh grid and Bayesian optimization methods. We completed 50 optimizations with different starting values for each participant and model, to ensure the obtained estimates were not a result of a local minimum. We took the maximum of all the runs as our estimate of the maximum-likelihood, and the corresponding parameter combination as our ML parameter estimates.

To validate the data-generating and model-fitting code, we performed parameter and model recovery. We simulated data from each model then fit each model to the simulated data. Successful parameter recovery occurs when the estimated parameters for the model that generated the data are equivalent or close to the true parameters. Parameter recovery is necessary for the interpretability of the parameter estimates. Successful model recovery occurs when the model which generated the data also fits the data better than any other model. Model recovery is necessary to ensure the models are distinguishable in a psychologically plausible model space. We successfully recovered both the parameters and models for each model.

### Code Availability

All analyses were conducted in MATLAB. Code used to fit and compare models and generate figures are available on the following github repository: github.com/aspenyoo/WM_resource_allocation. Code used to process, visualize, and score eye-tracking data are available on the Curtislab github repository: github.com/clayspacelab/iEye.

## Electronic supplementary material

Supplementary Information

## Data Availability

The datasets generated and analyzed during the current study are available in the data/priority directory of following github repository: github.com/aspenyoo/WM_resource_allocation.
