# Supplementary material for: Strategic allocation of working memory resource
Source: Sci Rep. 2018 Nov 1;8:16162. doi: 10.1038/s41598-018-34282-1 (PMC6212458; doi:10.1038/s41598-018-34282-1)
Supplement: Supplementary file 1 — Supplementary Information [file 41598_2018_34282_MOESM1_ESM.pdf]

Supplementary Information for
Strategic allocation of working memory resource

Aspen H. Yoo[1,2], Zuzanna Klyszejko[1,2], Clayton E. Curtis[1,2], Wei Ji Ma[1,2]
Department of Psychology, New York University, New York, NY, USA[1]
Center for Neural Science, New York University, New York, NY, USA[2]

Experiment 1

Model prediction

The goal of this section is to derive model predictions for estimation responses in Experiment 1. We model target location $\mathbf{s}$ as a two-dimensional vector corresponding to the target's horizontal and vertical coordinates. We denote the observer's estimate (saccade endpoint) of $\mathbf{s}$ by $\mathbf{x}$. We assume $\mathbf{x}$ follows a two-dimensional Gaussian distribution with mean $\mathbf{s}$ and covariance matrix $\frac{1}{J}\mathbf{I}$, where J is a scalar. This means that the magnitude of the estimation error, $\varepsilon \equiv ||\mathbf{x}-\mathbf{s}||$, follows a Rayleigh distribution with parameter $\frac{1}{\sqrt{J}}$.

Adopting a common formulation of the variable-precision model (van den Berg et al., 2012), we assume that precision, J, is itself a random variable that follows a gamma distribution with mean $\bar{J}$ and scale parameter τ. Our extension of the model allows the priority-specific $\bar{J}$ to vary; τ is fixed across conditions. We denote the mean total amount of available resource, the sum of the priority-specific precisions, as $\bar{J}_{\text{total}}$.

Resource allocation strategies

In Experiment 1, we test three models: the Proportional, Flexible, and Minimizing Error model. The models differ in how resource is allocated amongst the different items. We denote the proportion allocated to the high, medium, and low item as p_{high}, p_{med}, and p_{low}, respectively.

In the Proportional model, observers allocate resources equivalently to the experimental probe probabilities, i.e. $p_{\text{high}} = 0.6$, $p_{\text{med}} = 0.3$, $p_{\text{low}} = 0.1$. Its two free parameters are total resources $\bar{J}_{\text{total}}$ and scale parameter τ. In the Flexible model, the proportions allocated to each priority condition are fitted as free parameters. Thus, this model makes no hypothesis about how observers are allocated resource, only serves to describe what they do. Its free parameters are then $\bar{J}_{\text{total}}$, τ, p_{high}, and p_{med}.

The Minimizing Error model is a normative model in which the observer allocates resources in order to minimize expected behavioral cost across the experiment. We assume that the cost on a single trial is related to the magnitude ε

of the estimation error on that trial through a power law:

$$C_{\text{estimation}}(\varepsilon) = \varepsilon^{\gamma},$$

where $\gamma > 0$. Suppose now that on a given trial, the observer has allocated mean resource $\bar{J}$ to the probed stimulus. The expected cost on that trial is then an average over the errors ε that could occur on that trial:

$$\begin{aligned}
\bar{C}_{\text{estimation}}(\bar{J}) &\equiv \mathbb{E}(C_{\text{estimation}}|\bar{J}) \\
&= \int \varepsilon^{\gamma} p(\varepsilon|\bar{J}) d\varepsilon \\
&= \int \varepsilon^{\gamma} \int p(\varepsilon|J) p(J|\bar{J}) dJ d\varepsilon.
\end{aligned}$$

We now substitute the Rayleigh distribution for $p(\varepsilon|J)$ and the gamma distribution for $p(J|\bar{J})$ to evaluate this expression, where we write $k \equiv \frac{\bar{J}}{\tau}$:

$$\begin{aligned}
\bar{C}_{\text{estimation}}(\bar{J}) &= \int \varepsilon^{\gamma} \int \varepsilon J e^{-\frac{\varepsilon^2 J}{2}} \cdot \frac{1}{\Gamma(k)\tau^k} J^{k-1} e^{-\frac{J}{\tau}} dJ d\varepsilon \\
&= \frac{1}{\Gamma(k)\tau^k} \int \left(\int \varepsilon^{\gamma+1} e^{-\frac{\varepsilon^2 J}{2}} d\varepsilon \right) J^k e^{-\frac{J}{\tau}} dJ \\
&= \frac{\Gamma\left(\frac{\gamma+2}{2}\right)}{2\Gamma(k)\tau^k} \int \left(\frac{2}{J}\right)^{\frac{\gamma+2}{2}} J^k e^{-\frac{J}{\tau}} dJ \\
&= \frac{\Gamma\left(\frac{\gamma+2}{2}\right) 2^{\frac{\gamma+2}{2}}}{2\Gamma(k)\tau^k} \int J^{k-\frac{\gamma}{2}-1} e^{-\frac{J}{\tau}} \\
&= \frac{\Gamma\left(\frac{\gamma+2}{2}\right) 2^{\frac{\gamma+2}{2}}}{2\Gamma(k)\tau^k} \Gamma\left(k - \frac{\gamma}{2}\right) \tau^{k-\frac{\gamma}{2}} \\
&= \frac{\Gamma\left(\frac{\gamma+2}{2}\right) \Gamma\left(k - \frac{\gamma}{2}\right)}{\Gamma(k)} \left(\frac{2}{\tau}\right)^{\frac{\gamma}{2}},
\end{aligned}$$

where we assumed that $\gamma < 2k$.

So far, we have considered a trial with a given $\bar{J}$. Now, we ask how, for a given $\bar{J}_{\text{total}}$, τ, and γ, the observer should set p_{high}, p_{med}, and p_{low} to minimize the expected cost across the entire experiment. We refer to this expected cost as the "overall expected cost" (OEC); it is equal to

$$\text{OEC}(p_{\text{high}}, p_{\text{med}}, p_{\text{low}}) = 0.6\bar{C}(p_{\text{high}} \cdot \bar{J}_{\text{total}}) + 0.3\bar{C}(p_{\text{med}} \cdot \bar{J}_{\text{total}}) + 0.1\bar{C}(p_{\text{low}} \cdot \bar{J}_{\text{total}}).$$

We denote the resulting cost-minimizing proportions by p^*_{high}, p^*_{med}, and p^*_{low}. Each of these is a function of $\bar{J}_{\text{total}}$, τ, and γ.

Estimation of p^*_{high}, p^*_{med}, and p^*_{low}. We assume the observer calculates and uses these cost-minimizing proportions. While the brain may be able to do this optimization in a way we do not even begin to try to answer, we find the values of p^*_{high}, p^*_{med}, and p^*_{low} with fmincon in MATLAB's Optimization Toolbox (MathWorks). We begin the optimization from ten different starting points, to lower the probability of finding a local minimum, and choose the proportions corresponding to the lowest OEC. Note that this optimization is different from the optimization completed to estimate the ML parameters (explained below): the former is necessary to calculate the log-likelihood of a single parameter combination, and is thus completed thousands of times within one ML parameter estimation.

Maximum-likelihood parameter estimation

For each participant and each model, we estimated the parameters using maximum-likelihood estimation. The likelihood of the parameter combination θ for a given trial is defined as $p(\text{data}|\text{model},\theta)$. In Experiment 1, the only data is the saccade landing, $\hat{\mathbf{s}}$.

$$\begin{aligned}
p(\text{data} \mid \text{model},\theta) &= p(\hat{\mathbf{s}} \mid \text{model},\theta) \\
&= \iint p(\hat{\mathbf{s}} \mid \mathbf{x})p(\mathbf{x} \mid \mathbf{s},J)d\mathbf{x}\; p(\mathbf{s})p(J \mid \bar{J},\tau)dJ \\
&= \iint \delta(\hat{\mathbf{s}} - \mathbf{x})\mathcal{N}\left(\mathbf{x};\mathbf{s},\frac{1}{J}\mathbf{I}\right)d\mathbf{x}\; p(\mathbf{s})p(J \mid \bar{J},\tau)dJ \\
&= \int \mathcal{N}\left(\hat{\mathbf{s}};\mathbf{s},\frac{1}{J}\mathbf{I}\right)p(\mathbf{s})p(J \mid \bar{J},\tau)dJ \\
&\propto \int_{\mathbf{s}} \mathcal{N}\left(\hat{\mathbf{s}};\mathbf{s},\frac{1}{J}\mathbf{I}\right)p(J \mid \bar{J},\tau)dJ \\
&= \int_{\mathbf{s}} \text{Rayleigh}\left(\varepsilon;\frac{1}{J}\mathbf{I}\right)p(J \mid \bar{J},\tau)dJ
\end{aligned}$$

Notice that $\iint p(\hat{\mathbf{s}} \mid \mathbf{x})p(\mathbf{x} \mid \mathbf{s},J)d\mathbf{x}\; p(\mathbf{s})p(J \mid \bar{J},\tau)dJ$ can be simplified to $\int_{\mathbf{s}} p(\varepsilon \mid J)p(J \mid \bar{J},\tau)dJ$. We numerically integrate over J with 500 equally spaced bins. To calculate the log-likelihood, we take the logarithm of this value. To calculate the log-likelihood of a particular parameter combination θ, we sum the logs of the likelihoods across trials.

We used the optimization algorithm Bayesian Adaptive Direct Search (BADS; Acerbi & Ma, 2017) in MATLAB, which combines mesh grid and Bayesian optimization methods. We completed 50 optimization runs with different starting values for each participant and model, to lower the probability of a local minimum. We took the minimum negative log-likelihood of all the runs as our estimate of the maximum-likelihood, and the corresponding parameter combination as our ML parameter estimate.

Experiment 2

Model prediction

In Experiment 2, we model the memory estimation as described in Experiment 1. The goal of this section is to derive model predictions for the additional behavioral data: the circle wager.

We assume that on every trial, the observer chooses a circle radius r noisily around the value that maximizes the expected utility (EU) of that trial. The EU is calculated as the product between the utility of setting a circle with radius r and the probability that the true stimulus lies within the circle bounds (i.e., a hit). The observer calculates the utility as the number of points awarded for circle radius r raised to a power α that accounts for risk preferences, $120e^{-r\alpha}$. An $\alpha > 1$ corresponds to risk-seeking behavior (corresponding to smaller circles on average), while an $\alpha < 1$ corresponds to risk-averse behavior (corresponding to larger circles on average).

The probability of a hit is equivalent to the bounded integral of the posterior $p(\mathbf{s}|\mathbf{x})$ over the region described by the circle. For a two-dimensional Gaussian distribution, this is equivalent to a cumulative Rayleigh distribution evaluated

at r:

$$p_{\text{hit}}(r,J) \equiv p(\varepsilon \leq r|J) = \left(1 - e^{-\frac{r^2 J}{2}}\right).$$

We assume that the observer's decision noise follows a softmax rule, such that the probability of choosing r is

$$\begin{aligned} p(r|J) &\propto \exp\left(\beta \text{EU}(r,J)\right) \\ &= \exp\left(\beta \cdot \text{utility}(r) \cdot p_{\text{hit}}(r,J)\right) \\ &= \exp\left(\beta \cdot 120 e^{-r\alpha} \left(1 - e^{-\frac{r^2 J}{2}}\right)\right). \end{aligned}$$

Here, β, the inverse temperature parameter, controls the decision noise level: a lower β corresponds to more decision noise.

Resource allocation strategies

In Experiment 2, we test four models: the Proportional, Flexible, Minimizing Error, and Maximizing Points models. In the Proportional model, observers allocate resources equivalently to the experimental probe probabilities, i.e. $p_{\text{high}} = 0.6, p_{\text{med}} = 0.3, p_{\text{low}} = 0.1$. Its four free parameters are total resources $\bar{J}_{\text{total}}$, scale parameter τ, risk preference α, and inverse noise temperature β. In the Flexible model, the proportions allocated to each priority condition are fit as free parameters. Its six free parameters are then $\bar{J}_{\text{total}}$, τ, α, β, p_{high}, p_{med}. In the Minimizing Error model, observers allocate resource in order to minimize expected behavioral loss across the experiment exactly as described in Experiment 1. In Experiment 2, this strategy is a myopic: the observer does not take into account the subsequent decision they must make, but first maximizes performance in terms of estimation error, then maximizes EU. Its five free parameters are $\bar{J}_{\text{total}}$, τ, γ, α, and β. The optimal resource allocations $p^*_{\text{high}}, p^*_{\text{med}}, p^*_{\text{low}}$ depend on parameters $\bar{J}_{\text{total}}$, τ, and γ.

While observers in all models maximize the EU on every trial for a given J, the Maximizing Points model observer additionally allocates resources in order to maximize the expected utility across the entire experiment. We define the cost of a single trial as the negative EU on that trial:

$$C_{\text{wager}}(r|J) \equiv -\left(1 - e^{-\frac{r^2 J}{2}}\right) \cdot 120 e^{-r\alpha}.$$

The expected cost on that trial, for a given J, is an average of the costs for all possible radii r reported on that trial. However, J itself is a random variable, drawn from a distribution determined by a priority-specific $\bar{J}$. Thus, we must also marginalize over J to calculate the expected cost of a trial in each priority condition:

$$\begin{aligned} \bar{C}_{\text{wager}}(\bar{J}) &\equiv \mathbb{E}(C_{\text{wager}}|r,J) \\ &= \int C_{\text{wager}}(r|\bar{J}) p(r|J) dr \\ &= \iint -\text{EU}(r,J) p(r|J) p\left(J\,|\,\bar{J},\tau\right) dr dJ \end{aligned}$$

We numerically integrated over r and J to obtain the $\bar{C}_{\text{wager}}$ for a given $\bar{J}$. The OEC for this experiment is thus:

$$\text{OEC}(p_{\text{high}}, p_{\text{med}}, p_{\text{low}}) = 0.6\bar{C}(p_{\text{high}} \cdot \bar{J}_{\text{total}}) + 0.3\bar{C}(p_{\text{med}} \cdot \bar{J}_{\text{total}}) + 0.1\bar{C}(p_{\text{low}} \cdot \bar{J}_{\text{total}}).$$

In the Maximizing Points model, the cost-minimizing proportions $p^*_{\text{high}}, p^*_{\text{med}}$, and p^*_{low} are a function of all parameters $\bar{J}_{\text{total}}$, τ, α, and β. We obtain these values through the optimization methods described in Experiment 1.

Maximum-likelihood parameter estimation

Again, for each model and participants, we estimated the parameters that maximized the log-likelihood of the data given the model parameters. For Experiment 2, data consists of both the memory estimation and the post-decision wager. Thus, the likelihood depends on r in addition to $\hat{\mathbf{s}}$. In our calculation of the log-likelihood, we assume these measures are independent variables.

$$\begin{aligned} p(\text{data} \mid \text{model}, \theta) &= p(\hat{\mathbf{s}}, r \mid \text{model}, \theta) \\ &= \iint p(\hat{\mathbf{s}} \mid \mathbf{x}) p(\mathbf{x} \mid \mathbf{s}, J) d\mathbf{x}\; p(\mathbf{s}) p(r \mid J) p(J \mid \bar{J}, \tau) dJ \\ &= \int p(\hat{\mathbf{s}} \mid \mathbf{s}, J) p(\mathbf{s}) p(r \mid J) p(J \mid \bar{J}, \tau) dJ \\ &\propto \int_{\mathbf{s}} p(\hat{\mathbf{s}} \mid \mathbf{s}, J) p(r \mid J) p(J \mid \bar{J}, \tau) dJ \end{aligned}$$

We used the same optimization method as described in Experiment 1.

Optimal resource allocation

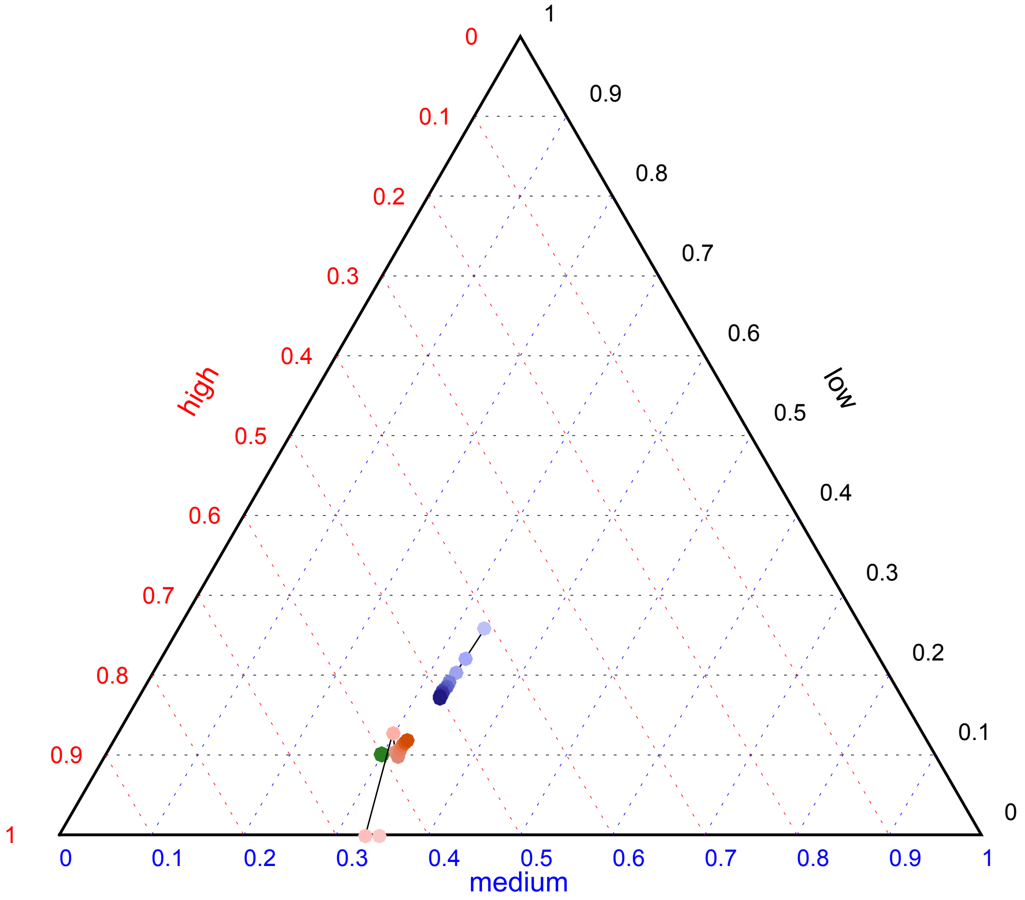

Figure 1: Optimal resource allocation as a function of total resource, $\bar{J}_{\text{total}}$ for different models (indigo: Minimizing Error, orange: Maximizing Points, green: Proportional). Each side of the triangle corresponds to the probe probability of or proportion allocated to each priority condition. With a lower $\bar{J}_{\text{total}}$, indicated by a lighter color, the models make very different predictions. The Minimizing Error model predicts more equal allocation than proportional to the probe probability, while the Maximizing Points model predicts dropping the low-priority target. As $\bar{J}_{\text{total}}$ increases, both models predict an allocation closer to the experimental probe probabilities. The prediction for the Proportional model is always the same as the experimental probe probabilities. Note that this plot was created by varying $\bar{J}_{\text{total}}$ from 1 to 10, and keeping $\tau = 0.4, \alpha = 1, \beta = 1$, and $\gamma = 1$.

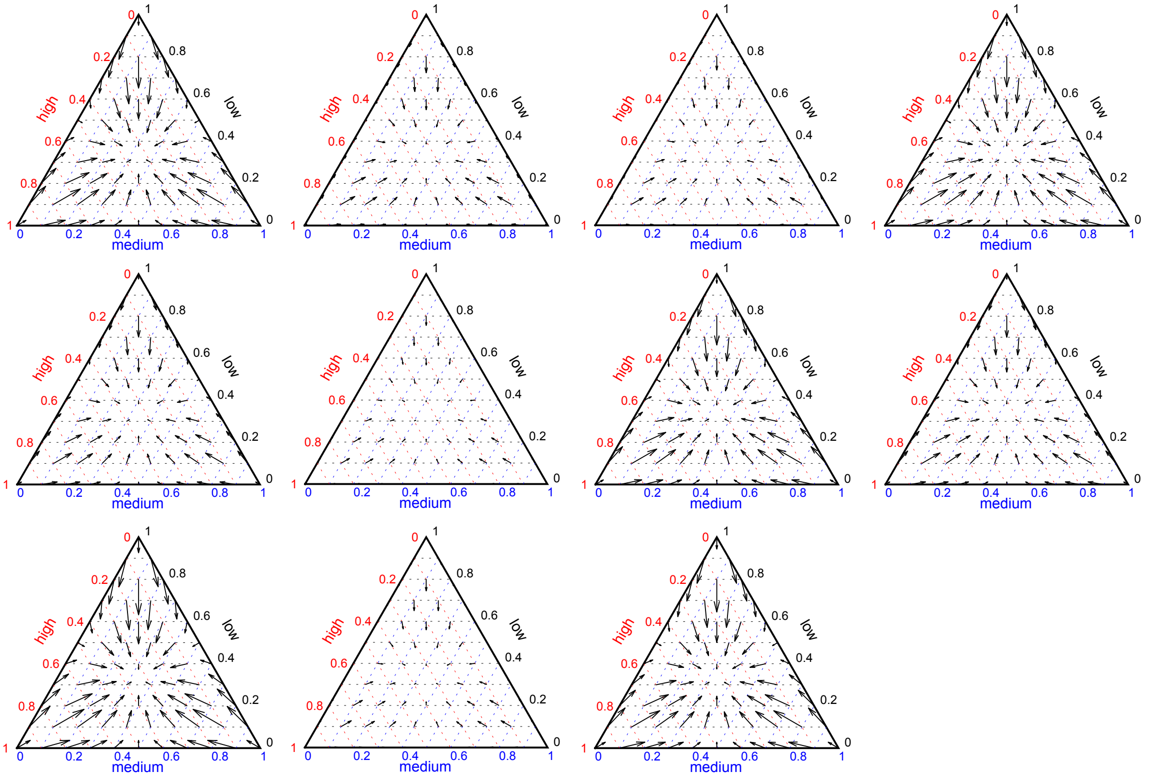

Figure 2: Optimal proportion allocation (tip of arrow) as a function of experimental probe probability (base of arrow) for the Minimizing Error model, for each participant in Experiment 2. Each side of the triangle corresponds to the probe probability of or proportion allocated to each priority condition. The Minimizing Error model predicts that the optimal allocation is more equal than the experimental probe probabilities, indicated by the arrows pointing toward the center of the triangle. .

Permutation test

In Experiment 2, we found a positive correlation between error magnitude and the radius of the circle wager. We believe this correlation was driven by a knowledge of internal noise, but it is possible that it is driven by a knowledge of stimulus-dependent noise. For example, in orientation perception, targets with orientations closer to the cardinal axis are perceived more accurately than obliquely-oriented objects (Girshick, Landy, & Simoncelli, 2011). We considered that there was a similar effect for memories of locations; perhaps objects closer to the cardinal axes are remembered with a different fidelity than those farther away. We did a regression to see if there was a trend between distance from cardinal axis (up to 45°) and estimation error. The oblique effect in memory of locations of objects was inconclusive. For seven of eleven participants, stimulus location did not significantly predict error ($p > 0.05$), but the remaining four participants had greater error when moving farther from the cardinal axes (M $\pm$ SEM regression weights: $1.40 \pm 0.84, p < 0.05$).

Because there is not a clear relationship between stimulus value and error, we cannot simply regress out any relationship between the two. However, there may still be some stimulus-dependent relationship that can still be driving the correlation between error and circle size. We decided to conduct a permutation test, which allows us to investigate this question without needing to describe or parameterize the relationship between the stimulus location and error. This permutation test followed the following steps for each participant and priority condition:

1. Bin error and circle size data according the angular distance of the target from the horizontal axis (10° to 80° in 10° increments. Note that 10°, 170°, 190°, and 350° are all 10° away from horizontal axis)

2. Permute circle size within each bin

3. Combine bins

4. Compute correlation between error and circle size Repeat steps 2 through 4 a thousand times

In step one, we combined data according to their angular distance from horizontal in an effort to increase the number of trials per bin. This grouping assumes that the main stimulus-dependent noise would be relative to the cardinal axes, not any hemispheric differences. In an ideal scenario, we would then be able to conduct a correlation within each bin. However, there were as few as two trials in one bin, so computing a correlation for each bin was not feasible.

For step two, we performed a special type of permutation called a derangement, in which no element is placed its original location. We conducted a derangement because it is more robust to small sample sizes than a regular permutation. For example, in a regular permutation of two data points, half of the time you would get the original configuration, leading to biased results.

By completing permutations on multiple small bins within each dataset, the recombined, permuted dataset maintains any correlations that are stimulus-location driven, while removing any relationship driven by a knowledge of internal memory fluctuations. Therefore, if the correlation was largely due to the stimulus location, then the correlation of the permuted data would still be positive. If, on the other hand, the correlation was driven by internal fluctuations that were independent of the location of the stimulus, the positive correlation observed in the non-permuted data would be significantly reduced in the permuted data.

To test if the true correlation was significantly higher than the null correlations, we conducted a Wilcoxon signed-rank test between the medians of each null correlation distribution (for each priority and subject) and the respective true correlations. We found that the actual correlations ($M \pm SEM : 0.29 \pm 0.04$) were significantly higher than the median of the correlations obtained in the null distribution ($M \pm SEM : -0.007 \pm 0.006; z = -4.69, p < 1e-5$), suggesting that the correlation within each priority condition was driven by internal fluctuations in the quality of the memory representation above and beyond any location-dependent variation.

We additionally investigated whether the observed correlation between error and circle size could be explained by knowledge of delay. Like before, we first tested if there was a significant change in error as a function of delay. Delay did not significantly predict error for nine of eleven participants ($p > 0.05$), and predicted an 0.10 and 0.11 dva increase

in error for every second increase in delay for the other two participants ($p < 0.01$). We completed a permutation test, in which we binned data by participant, priority, and delay time (1 to 4 seconds in 0.5 second increments); deranged the circle sizes within each bin; combined data across delay bins; then computed the correlation between error and circle size, resulting in a correlation for each participant and priority condition. We repeated this process 1000 times, to get a null distribution of correlation coefficients. Like before, we conducted a Wilcoxon signed-rank test between the medians of each null correlation distribution (one for each priority and subject) and the respective true correlations. We found that the actual correlations ($M \pm SEM : 0.29 \pm 0.04$) were significantly higher than the median of the correlations obtained in the null distribution ($M \pm SEM : -0.004 \pm 0.004; z = -4.53, p < 1e-5$), suggesting that the correlation within each priority condition was driven by internal fluctuations in the quality of the memory representation above and beyond any delay-dependent variation.
